# A Case of Hypertrophy of the Spleen and Post Mortem Examination

**Published:** 1857-08

**Authors:** William H. Philpot

**Affiliations:** Redbone, Ga.


ATLANTA

Medical and Surgical Journal.

VOL. II.] AUGUST, 1857. [NO. 12

### ORIGINAL COMMUNICATIONS.

### ARTICLE I.

*A Case of Hypertrophy of the Spleen and Post Mortem examination.* By WILLIAM H. PHILPOT, M. D., of Redbone, Ga.

I WAS called the 20th June last, about 11 o'clock, at night, to the plantation of Cyrus Robinson, of this county, to see Salina, a bright mulatto woman some 30 or 35 years of age, who the messenger informed me was supposed to be dying. I found her suffering severe pains in the Epigastric, right and left Hypochondriac regions, and very severe, cephalalgia, and great dyspnea,—pulse feeble and some 80 beats. The patient was rational, but suffering so much pain I could ascertain but little as regards her condition. She would point me to her head as the most painful. I gave her a mixture of Tincture Valerian and Sulp. Ether—blistered a small spot back of each ear with strong Aqua Ammonia, and applied some morphia, to the denuded surface, and after abstracting some 8 or 10 ℥ of blood from the arm, she was soon asleep.

The lady, the wife of the overseer, then informed me that "Salina was taken sick nearly two years ago with chills and fever, and in a few days a swelling commenced in her left side. She has been under the treatment of several respectable physicians for Enlargement of the Spleen; that after taking physic the most of the time, up to a month or so back, it was decided nothing more could be done, and she had suffered at times as

I then saw her. She was daily expected to die for the last month or two."

I saw Salina early next morning, she was easy the remainder of last night, and slept some little—was suffering a great deal with her head—great dyspnea and severe pains in the right and left Hypochondriac, and the Epigastric region—and is unable to move without assistance. I found, on examination, the left side of abdomen greatly tumified, as was found to be the Epigastric and right Hypochondriac region. Dullness was found to exist on percussion, over both, and the respiratory murmur absent in the left lung. She had frequent cough attended with a thin frothy expectoration mixed with mucus. Her Stomach was very irritable, and she had vomited up several clots of blood as large as my thumb. She seemed In Articulo Mortis from the great dyspnea.

Nothing I could administer internally seemed to do so well, or relieve the pain and dyspnea so quick as blood letting. Salina suffered until the morning of the 26th, about 4 o'clock, when she died.

Having to send some six or seven miles to procure professional assistance in performing the Post Mortem, and it then being very uncertain if the physician would be found at home, I commenced the examination alone, some six or eight hours after death. Owing to my being without assistance, and the fatigue and hurry, I did not extend the examination as far and as closely as I would have done or wished.

Upon making an incission, so as to expose the whole contents of the abdomen, the spleen was found occupying a position, extending from the diaphragm to the illiac region; its vessels very much enlarged and filled with blood, the upper portion or margin was found lying against the Cardiac extremity of the Stomach, while its concave surface was firmly pressed against it by the left lobe of the Liver. I removed the Spleen—it weighed 5 lbs. 4 or 5 ℥. I send it on for your College Museum.

The Liver was found of an enormous size, occupying the whole lumbar and Epigastric region, and with the spleen seeming nearly to fill the whole of the abdominal cavity, compressing the stomach and intestines against the spinal column. Firm adhesions had taken place between its peritoneal covering and the abdominal parietes and diaphragm, requiring some force to separate, and rupturing the membrane in its separation. The surface was rough and uneven, made so by lumps of various sizes, which I supposed to be Tubercles. Upon making an incission into the substance of this organ, some dark looking fluid was seen to flow out. The gall cyst was distended to some extent, and filled with dark colored bile. Adhesions were also found to exist between it and some portions of the stomach. This organ being very small and its coats thin and transparent—it was empty save some little dark brown fluid. The liver and spleen forced up the diaphragm into the thoracic cavity, enlarging the cavity of the abdomen, and of course reducing that of the thorax. I did not remove the liver from its connections, but from the enormous size, am satisfied it would have weighed some 12 or 15 lbs. The kidneys were somewhat enlarged and congested, but looked natural and healthy otherwise.

On cutting into and exposing the contents of the thorax, the Pericardium was found to contain some half pint or more of fluid. The lungs atrophied and covered with melanic deposits; the left one not a great deal larger than my hand, and extensive and firm adhesions, between it and the Pleura Costalis; the right lung was larger—was melanotic and but little if any adhesions. I did not, owing to time and circumstances, examine the brain.

Thus I have endeavored to give you as correct an account as possible of my hasty and hurried examination.

While I am writing, let me give you a rare case of Midwifery that occurred in my neighborhood a few weeks ago.

Nancy, a negro woman, pregnant, belonging to Mr. J. P. Lenard, started off on a tramp on Sunday to see some of her dark friends on a neighboring plantation. When some four miles from home, labor commenced, and the pains increasing so fast and strong, she could not proceed any farther, and in a fence corner, some four miles from home, without any assistance, she gave birth to a large fine child, and *after a little* she "*broke*" the cord, and, with it in her fingers, wrapped the little fellow up in some of her own clothing and started home, was overtaken by a heavy rain and walked in it nearly all the way.

An old negro mid-wife on the plantation made an examination, and not finding the placenta, informed the husband of the girl, that if the after-birth was left on the ground and rotted, that his wife would gradually sink away and die. Off he starts on a hunting expedition, taking his wife's track back; but could find nothing of the object looked for, and returned home with a sad and disconsolate heart, but soon was comforted by the old granny who told him, the after-birth was eaten by bugs which would save the life of his wife.

Nancy and the baby did well; she being up and about in a few days without any unpleasant symptoms.

